# Long-term growth data of *Escherichia coli* at a single-cell level

**DOI:** 10.1038/sdata.2017.36

**Published:** 2017-03-28

**Authors:** Yu Tanouchi, Anand Pai, Heungwon Park, Shuqiang Huang, Nicolas E. Buchler, Lingchong You

**Affiliations:** 1 Department of Bioengineering, Stanford University, Stanford, California 94305, USA; 2 The Gladstone Institutes (Virology and Immunology), San Francisco, San Francisco, California 94107, USA; 3 Fred Hutchinson Cancer Research Center, Seattle, Washington 98109, USA; 4 Department of Biomedical Engineering, Duke University, Durham, North Carolina 27708, USA; 5 Department of Physics, Duke University, Durham, North Carolina 27708, USA; 6 Department of Biology, Duke University, Durham, North Carolina 27708, USA; 7 Center for Genomic and Computational Biology, Duke University, Durham, North Carolina 27708, USA; 8 Department of Molecular Genetics and Microbiology, Duke University Medical Center, Durham, North Carolina 27710, USA

**Keywords:** Time-lapse imaging, Escherichia coli, Microbiology, Systems biology, Time-lapse imaging

## Abstract

Long-term, single-cell measurement of bacterial growth is extremely valuable information, particularly in the study of homeostatic aspects such as cell-size and growth rate control. Such measurement has recently become possible due to the development of microfluidic technology. Here we present data from single-cell measurements of *Escherichia coli* growth over 70 generations obtained for three different growth conditions. The data were recorded every minute, and contain time course data of cell length and fluorescent intensity of constitutively expressed yellow fluorescent protein.

## Background & Summary

Cell-size homeostasis is a fundamental aspect in biology. During bacterial growth, a cell approximately doubles in size before it divides to produce two daughter cells. Although this process is subjected to the inherent stochasticity of cellular processes (known as cellular noise), cells appear to maintain their approximate cell size. The underlying control mechanism and the dynamics of cell size remain poorly understood due to the lack of experimental tools to measure cell size of individual bacterium over long periods of time in a high-throughput manner. Recent advancement in microfluidic technology provide a solution to this problem, enabling cell-size measurements of hundreds of single bacterial cells over more than one hundred generations^1^.

Using a microfluidic device called mother machine, we recently characterized long-term cell-size dynamics in *Escherichia coli*^2^. By analysing about 280 cell lineages over 70 generations (~20,000 cell cycles), we found that cell size at birth could show a transient oscillation with various periods, which can be longer than 15 generations. Further analysis revealed that cell size at division is a linear function of cell size at birth with some stochasticity, which we call a ‘noisy linear map’. Using mathematical modelling we showed that the noisy linear map can explain the observed transient oscillations in cell size. Moreover, we demonstrated that in addition to cell size, gene expression-even when it is constitutively expressed-could also show transient oscillations with periods spanning multiple cell cycles. We confirmed this observation with other data sets that had previously been published by other groups. In particular, the noisy linear map was observed in 10 different experimental conditions, covering different growth media, temperatures, *E. coli* cell strains as well as another rod-shape bacterium, *Bacillus subtilis* and fission yeast *Schizosaccharomyces pombe*. In this Data Descriptor, we provide a detailed description of the *E. coli* data set, which contains time course data of cell size, cell division time, and gene expression (measured as fluorescence intensity of constitutively expressed yellow fluorescent protein) obtained under three different temperatures.

## Methods

The following sections are an expanded version of the methods description provided in Tanouchi *et al.*^2^.

### Fabrication of microfluidic device

The mother machine was fabricated according to the previously published procedure^1^ except that our mould was reverse-fabricated from the original mother machine device. This was achieved by pouring epoxy onto the original mother machine device (kind gift from Dr Jun). Replicas of the mother machine were then created by pouring PDMS onto this mould and solidifying the polymer at 80 °C for 30 min. The input and output ports, both about 0.75 mm in diameter, were created by using biopsy punches. The PDMS device was then washed with pentane to remove any polymer residue. Lastly, the resulting PDMS device was then bonded to a glass cover slip by plasma treatment. Prior to loading cells, the device was cleaned using 70% ethanol, washed twice with distilled water, and all liquid was then expunged with air.

### Cell strain and growth condition

An *E. coli* strain MC4100 that constitutively expresses YFP (*galK::P*_*lac*_*-yfp amp*^*R*^; kind gift from Dr Kishony^3^) was used in all experiments. We followed a similar procedure as described in the previous study^1^ for long-term imaging of cells in the mother machine. An overnight culture grown in LB at 37 °C was diluted 100 fold in 5 ml fresh LB and grown at 37 °C. After 8 h, 500 μl of the culture was spun down in a 1.5 ml microcentrifuge tube, and the supernatant was removed by inverting the tube. The pellet was then resuspended in the remaining volume (typically 200–250 μl), and the cells were loaded into the mother machine using a syringe. After the loading, the device was spun for three minutes using a mini centrifuge to promote cell trapping in the side channels of the device. Fresh LB was introduced to remove cells not trapped and a continuous flow (100 μl/h) was maintained. We waited for at least 2 h with continuous medium flow before image acquisition. Throughout the experiment, carbenicillin (50 μg/ml) was added in growth medium.

### Image acquisition

Images were acquired every one minute using DeltaVision Elite microscope (Applied Precision) with a motorized stage and an Evolve EM-CCD camera (Photometrics) with either 100x DIC objective or 60x phase objective. When 60x phase objective was used, additional x2 auxiliary magnification was also used. The microscope and its growth chamber were equilibrated at an appropriate temperature (37, 27, or 25 °C) prior to the experiment, and the temperature was maintained throughout the experiment.

### Image analysis and data processing

We developed custom codes in C++, FIJI and Matlab for image segmentation and analysis. First, we detected boundaries between cells based on rescaled fluorescent images by finding minima of fluorescence intensity along the channel direction of the mother machine. Each image was first filtered using a 3-by-3 median filter, and fluorescent values were rescaled so that top and bottom 2% become saturated in the dynamic range. Erroneous boundaries (e.g., mother and daughter cells merged after division) were checked manually and corrected for all mother cells. Once the cell boundaries were determined, cell masks were generated by applying threshold (relative to maximum fluorescence value) on fluorescence intensity. Cell length was calculated by computing the major axis length of the mask. The average fluorescence intensity was also obtained using the masks. After segmentation, cell divisions were detected based on the change in cell length. Division events were identifiable in the data sets as a clear and large drop in the cell size. Also, we assumed cell divisions to be at least 10 min apart. The data resulting from this section can be found in MC4100_37C.zip, MC4100_27C.zip, and MC4100_25C.zip [Data Citation 1].

In our previous publication^2^, we selected cell lineages that contained measurements of full 70 generations. Also, we considered cells were filamentous when (1) initial cell length was larger than $\bar{L}+2\sigma_{L}$ ($\bar{L}$ and *σ*_*L*_ are the average and s.d. of the cell size distribution, respectively) or (2) final cell length was larger than $2\left(\bar{L}+2\sigma_{L}\right)$. We excluded these instances in some analysis. In addition, we excluded cell cycles whose initial cell length was smaller than $\bar{L}-2\sigma_{L}$. We only found one such instance in our data set. These procedures filtered out ~0.6% of total cell cycles.

### Code availability

The custom codes used for image processing are available upon request.

## Data Records

Three microscope experiments were performed under three different growth temperatures (37, 27, or 25 °C), and images were analysed as described in the methods section above. Each data record corresponds to a single experiment, and for each experiment there are multiple data sets associated with each mother cell lineages. Each data set includes microscope image file (e.g., xy01_01.tif) and analysis data (e.g., xy01_01.txt) containing time, division flag (1 if division occurred), cell length, per-cell total fluorescence intensity, and average fluorescence intensity of the mother cell. The numbers in the file name indicate the field of view and mother cell number within it, respectively. We also included sample images for each data record. Please refer to Table 1 for the description of each file on figshare.

### Data record 1

This data record contains data sets of mother cells grown at 37 °C. There are a total of 160 mother cell lineages.

### Data record 2

This data record contains data sets of mother cells grown at 27 °C. There are a total of 54 mother cell lineages.

### Data record 3

This data record contains data sets of mother cells grown at 25 °C. There are a total of 65 mother cell lineages.

## Technical Validation

### Example time course of cell growth and fluorescence intensity

**Figure 1:**
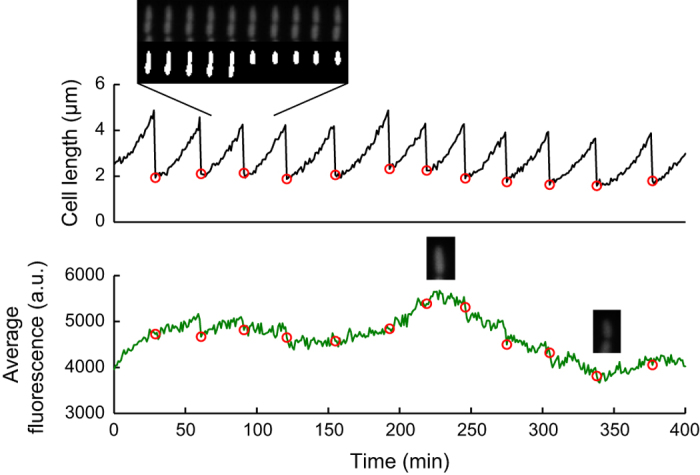
An example time course of cell size (top) and mean fluorescence intensity (bottom) from the experiment performed at 37 °C. Sample microscope images and their segmentation masks are also shown. Red circles indicate cell division.

Figure 1 shows an example time course of one of the mother cell lineages from the experiment performed at 37 °C. As expected from typical *E. coli* growth, the image analysis generated distinct cycles of cell elongation and division (Fig. 1, top). The average fluorescence intensity changes smoothly and appears to fluctuate with a longer time-scale than cell cycle (Fig. 1, bottom; see ref. 2 for further analysis on this aspect). The difference between high and low fluorescence intensities is visible in microscope images (Fig. 1, bottom, inset).

### Image analysis using two different thresholds produce consistent results

**Figure 2:**
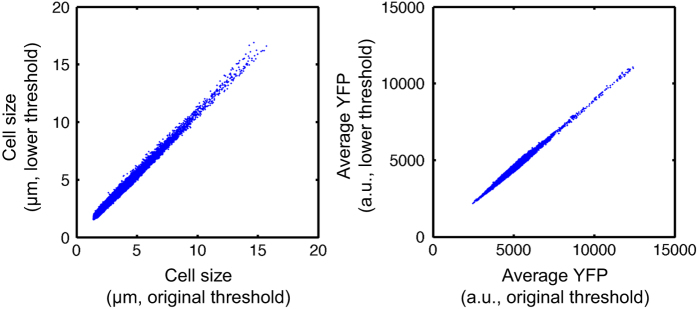
A comparison of image analysis using two different segmentation parameters. Images were re-analyzed using lower threshold value on fluorescence intensity, and the cell size (left) and mean fluorescence intensity (right) were compared. *R*^2^ values were 0.983 and 0.981 for cell length and average fluorescence intensity, respectively.

As described in the Methods section, cell masks were generated by applying threshold on fluorescence intensity. In theory, lowering the threshold will make the cell mask larger, resulting in larger cell length and lower average fluorescent intensity. However, as long as the threshold is within a reasonable range, different threshold should not cause qualitative change in our results. To examine this, we compared segmentation results from two different threshold values. Consistent with the notion above, lowering threshold resulted in overall larger cell length and lower average fluorescence intensity (Fig. 2). Importantly, the two segmentation results are linearly correlated with R^2^ close to 1 (0.983 for cell length and 0.981 for average fluorescence intensity), indicating that our results are qualitatively robust to the segmentation threshold.

### Comparison with other studies

As discussed in detail in our previous publication^2^, our data set was consistent with data sets from previous studies^1,4^. In particular, the linearity between initial and final cell sizes were consistently observed both in our data set and the data sets from previous studies^1,4,5^ despite different methods for image processing. This consistency indicates that the quality of our data set is comparable to that of previous studies.

## Additional Information

**How to cite this article:** Tanouchi, Y. *et al.* Long-term growth data of *Escherichia coli* at a single-cell level. *Sci. Data* 4:170036 doi: 10.1038/sdata.2017.36 (2017).

**Publisher’s note:** Springer Nature remains neutral with regard to jurisdictional claims in published maps and institutional affiliations.

## Supplementary Material



## Figures and Tables

**Table 1:** Description of files deposited in FigShare.

**Data record**	**File name**	**Description**
Data record 1	MC4100_37C.zip	A.zip file containing time-lapse microscope images (.tif) of 160 mother cell lineages cultured at 37C
	Analysis_MC4100_37C.zip	A.zip file containing analysis data files (.txt) of mother cells in MC4100_37C.zip (after manual correction of erroneous segmentations)
	MC4100_37C_Sample1.jpg	A sample image from MC4100_37C.zip
	MC4100_37C_Sample2.jpg	A sample image from MC4100_37C.zip
	MC4100_37C_Sample3.jpg	A sample image from MC4100_37C.zip
Data record 2	MC4100_27C.zip	A.zip file containing time-lapse microscope images (.tif) of 54 mother cell lineages cultured at 27C
	Analysis_MC4100_27C.zip	A.zip file containing analysis data files (.txt) of mother cells in MC4100_27C.zip (after manual correction of erroneous segmentations)
	MC4100_27C_Sample1.jpg	A sample image from MC4100_27C.zip
	MC4100_27C_Sample2.jpg	A sample image from MC4100_27C.zip
	MC4100_27C_Sample3.jpg	A sample image from MC4100_27C.zip
Data record 3	MC4100_25C.zip	A.zip file containing time-lapse microscope images (.tif) of 65 mother cell lineages cultured at 25C
	Analysis_MC4100_25C.zip	A.zip file containing analysis data files (.txt) of mother cells in MC4100_25C.zip (after manual correction of erroneous segmentations)
	MC4100_25C_Sample1.jpg	A sample image from MC4100_25C.zip
	MC4100_25C_Sample2.jpg	A sample image from MC4100_25C.zip
	MC4100_25C_Sample3.jpg	A sample image from MC4100_25C.zip
	NoteForAnalysisFiles.txt	Description of each column in the analysis data files
